# Political Regimes, Political Ideology, and Self-Rated Health in Europe: A Multilevel Analysis

**DOI:** 10.1371/journal.pone.0011711

**Published:** 2010-07-22

**Authors:** Tim Huijts, Jessica M. Perkins, S. V. Subramanian

**Affiliations:** 1 Department of Sociology, Radboud University Nijmegen, Nijmegen, The Netherlands; 2 Department of Health Policy, Harvard University, Cambridge, Massachusetts, United States of America; 3 Department of Society, Human Development and Health, Harvard School of Public Health, Boston, Massachusetts, United States of America; Erasmus University Rotterdam, Netherlands

## Abstract

**Background:**

Studies on political ideology and health have found associations between individual ideology and health as well as between ecological measures of political ideology and health. Individual ideology and aggregate measures such as political regimes, however, were never examined simultaneously.

**Methodology/Principal Findings:**

Using adjusted logistic multilevel models to analyze data on individuals from 29 European countries and Israel, we found that individual ideology and political regime are independently associated with self-rated health. Individuals with rightwing ideologies report better health than leftwing individuals. Respondents from Eastern Europe and former Soviet republics report poorer health than individuals from social democratic, liberal, Christian conservative, and former Mediterranean dictatorship countries. In contrast to individual ideology and political regimes, country level aggregations of individual ideology are not related to reporting poor health.

**Conclusions/Significance:**

This study shows that although both individual political ideology and contextual political regime are independently associated with individuals' self-rated health, individual political ideology appears to be more strongly associated with self-rated health than political regime.

## Introduction

The association between political ideology and health has been the subject of numerous studies in social epidemiology and political sociology.[1] These studies have mostly examined either the association between political ideology and health at the ecological level (e.g., countries or municipalities), or the contextual influence of political ideology on the health of individuals.[2], [3], [4], [5], [6] In general, average health appeared to be better in areas with conservative majorities and in countries with social democratic or liberal welfare regimes. However, such studies examining the association between political ideology and health at the aggregate level cannot provide insights into how individuals' political ideology might be associated with their health. Recently, three studies examining political ideology and health among people in the United States (US), Europe, and Japan demonstrated, separately, that individuals' political ideology is associated with their health status.[7], [8], [9] The lower poor health status among republicans and individuals with a rightwing self-placement could not be explained by the higher socioeconomic status (SES) of these groups in these studies. The studies suggested that individuals with conservative values may be less likely to engage in adverse health behavior.

The ideology of a country's political regime is influenced by the political ideology of its residents. Therefore, it is possible that part of the association between political regime and self-rated health found in earlier work is due to the relationship between individual political ideology and health. Similarly, the association between individual political ideology and health that was found in earlier studies may partly capture effects of political regimes on individuals' health. Moreover, political regimes and individuals' political ideology may also interact; similar to findings on the relationship between SES and health in earlier work,[1], [2] the association between individual ideology and health may be particularly strong under certain regime types and relatively weak under other types. In this analysis, we simultaneously examined the associations between self-rated health and measures of individual political ideology, political regimes, and country level aggregations of individual political ideology using data on Europe. To our knowledge, our contribution is the first to separate effects of individual political ideology and ecological measures of political ideology on health.

## Methods

### Data and Measures

The 2002, 2004, and 2006 European Social Survey (ESS) data, available for 29 European countries and Israel [10], [11], were pooled and analyzed. These data allow an examination of health status and political ideology at an individual level as well as offer the opportunity to examine this micro-level association under several political regime types. The ESS is generally considered to be a prominent source of cross-national data with strong validity and reliability, with a mean response rate of over 60% [10], [11].

Self-rated health was measured by asking respondents ‘*How is your health in general? Would you say it is very good, good, fair, bad, or very bad?*’ We converted the original measure into a binary variable with bad or very bad health (hereafter referred to as poor health)  = 1, 0 otherwise.

Political ideology at the individual level was based on a question, “*In politics, people sometimes talk of “left” and “right”. Where would you place yourself on this scale, where 0 means the left and 10 means the right?*” In addition to this linear measure of ideology, we also grouped the scale into ‘Left’ (21.9%) consisting of the first four categories, ‘Right’ (24.7%) containing the last four categories, and ‘Middle’ (53.4%), comprising the three middle categories, for ease of interpretation and presentation.

Two measures were used to indicate ecological political ideology. First, we distinguished six political regime types: social democratic (i.e., Denmark, Finland, Norway, and Sweden), Christian conservative (Austria, Belgium, France, Germany, Iceland, Italy, Luxembourg, Netherlands, and Switzerland), liberal (Ireland, Israel, and United Kingdom), former Mediterranean dictatorships (Cyprus, Greece, Portugal, and Spain), Eastern Europe (Bulgaria, Czech Republic, Hungary, Poland, Slovenia, and Slovakia), and former Soviet republics (Estonia, Latvia, Russian Federation, and Ukraine). This classification corresponds to typologies used in earlier studies on political regimes and health inequalities [2], [6]. A brief description of the political regime types in this study is provided in **Table 1** (for a more detailed description, we refer to earlier work by others) [2], [6]. Second, an aggregated measure of individual political ideology was obtained by computing the country-specific average score on the individual left-right self-placement measure. Whereas the political regime measure indicates the influence of political ideology on health through institutional mechanisms, the aggregated ideology measure reflects the political ideology of individuals in the respondent's living environment.

**Table 1:** Description of political regimes.[2], [6]

*Social Democratic* - These countries are characterized by high governmental redistribution of resources and high expenditure on social security. Social inequality is generally low. Reliance on family for support and health care, and on self-regulation, are low as compared to other political regimes.	*Liberal* - Countries with this political regime type rely strongly on self-regulation through the market. Expenditure on social security and governmental redistribution of resources, as well as reliance on family, are low in these countries. Earlier studies did not include Israel in this category, probably because of data unavailability.
*Christian conservative* - These countries take in a middle position between social democratic regimes and liberal regimes in terms of reliance on self-regulation and governmental intervention in redistribution of resources. Reliance on family for support and health care is stronger as compared to both social democratic and liberal regimes, but weaker in comparison with former Mediterranean dictatorships. Cooperation between progressive and conservative parties is relatively high. Earlier studies did not include Iceland in this category, probably because of data unavailability.	*Former Mediterranean dictatorships* - These countries are sometimes also labeled ‘ex-fascist’. They are characterized by low state intervention and low social security expenditure, and by strong reliance on family and other informal ties for support and health care. In some studies, Italy is included as well (in this case, the regime is labeled ‘Southern European’). In this study, we excluded Italy since this did not have a dictatorial regime in recent decades. Earlier studies did not include Cyprus in this category, probably because of data unavailability.
*Eastern Europe* - These countries have only recently been recognized as a distinct political regime type. During the last two decades, they have experienced a transition from communism to market-oriented political systems. Reliance on family and other informal ties for support and health care is low but increasing.	*Former Soviet Republics* - This political regime is inherently the same as Eastern Europe, but taken as a distinct category in our study because of the difference in the recent past between these countries and Eastern Europe. Although under communist regime, Eastern European countries to a certain extent retained their independence. The former Soviet republics only became independent in the early nineties, and have therefore experienced a stronger influence of communism and a more dramatic political past.

### Statistical Analysis

In all models, age, sex, survey year, years of full-time education, being in paid employment, and net household income in the last year prior to the survey were included as covariates. After exclusion of missing values on the outcome and independent variables, the final analytic sample consisted of 84 402 individuals. We used multilevel, binary logistic regression model procedures as implemented in MlwiN v 2.10 that took account of the hierarchical structure of the data (i.e., individuals nested within countries).

### Ethics Statement

The study was reviewed by Harvard School of Public Health Institutional Review Board and was considered as exempt from full review as the study was based on an anonymous public use data set with no identifiable information on the survey participants.

## Results

In **Table 2**, descriptive statistics for the variables in the analyses are presented, as well as the percentage reporting poor health by categories of independent variables. The distribution of the categorical measure of political ideology shows that the group of left-wing respondents and the group of right-wing individuals are about equal in size (21.9% and 24.7% respectively), with the middle category being largest (53.4%). Of the respondents who reported to have left-wing political views, 9.4% judged their health to be poor. For right-wing respondents and respondents in the middle of the ideological range, these percentages amount to 7.1% and 7.4% respectively. Comparing the political regimes, respondents from Social Democratic, Christian conservative, and liberal countries are in relatively good health (respectively, 4.8%, 5.9%, and 5.5% report poor health in these societies). Of the respondents in the former Soviet Republics, 18.2% report poor health. Percentages reporting poor health in the former Mediterranean dictatorships and Eastern Europe amount to 10.1% and 12.8% respectively.

**Table 2:** Descriptive statistics of all variables and percentage reporting poor health per category in the 2002/04/06 European Social Survey (n = 84 402).

Variables	Frequency	Percentage	Mean	Standard deviation	% Poor health
Age (18 = 0)			30.02	17.07	
Sex					
Male	40 970	51 5			6.7
Female	43 432	48 5			8.7
Survey year					
2002	28 400	33.6			7.4
2004	29 603	35.1			8.3
2006	26 399	31.3			7.5
Years of education			12.31	4.01	
Paid employment					
No	41 426	49.1			13.3
Yes	42 976	50.9			2.5
Household income			6.18	2.63	
Left-right self-placement			5.07	2.16	
Left-right self-placement (categorical)					
Left	18 479	21.9			9.4
Middle	45 111	53.4			7.4
Right	20 812	24.7			7.1
Political regime					
Social democratic	4	22.3			4.8
Christian conservative	9	34.7			5.9
Liberal	3	11.9			5.5
Former Mediterranean dictatorships	4	10.4			10.1
Eastern European	6	15.7			12.8
Former Soviet Republics	4	5.1			18.2
National average left-right self-placement score			5.07	0.35	

Note: ‘Left’ = 0–3 on the left-right self-placement scale, ‘Middle’ = 4–6, and ‘Right’ = 7–10. Frequencies with political regime indicate the number of countries instead of individuals.

**Table 3** reports the results for the models in which the continuous and categorical measures of individual political ideology were used. In Model 1, the association between individual political ideology (continuous) and self-rated health was estimated without accounting for ecological political ideology. For a unit increase in the political ideology scale (towards the right) the odds-ratio (OR) for reporting poor health decreased (OR 0.96, 95% confidence interval [CI] 0.95–0.97).

**Table 3:** Results of age-, sex-, and SES-adjusted binary logistic, multilevel models, displaying odds-ratios (OR) and 95% confidence intervals (CI) for reporting poor health by individual political ideology (continuous and categorical), political regime group, and aggregate political ideology in the 2002/04/06 European Social Survey (n = 84 402).

	Model 1		Model 2		Model 3		Model 4		Model 4(a)	
Variables	OR	95% CI	OR	95% CI	OR	95% CI	OR	95% CI	OR	95% CI
Left-right self-placement (LR)	0.96	(0.95, 0.97)					0.96	(0.94, 0.97)		
Left (ref.)									1.00	
Middle									0.81	(0.76, 0.87)
Right									0.73	(0.68, 0.80)
Social democratic (ref.)			1.00				1.00		1.00	
Christian conservative			1.07	(0.70, 1.63)			0.90	(0.57, 1.43)	0.90	(0.57, 1.43)
Liberal			0.99	(0.58, 1.70)			0.97	(0.58, 1.63)	0.97	(0.58, 1.63)
Former Mediterranean dictatorships			1.00	(0.61, 1.65)			0.88	(0.53, 1.45)	0.88	(0.53, 1.45)
Eastern Europe			1.66	(1.05, 2.61)			1.42	(0.88, 2.29)	1.41	(0.88, 2.29)
Former Soviet republics			2.29	(1.39, 3.78)			2.23	(1.37, 3.60)	2.23	(1.37, 3.60)
National average LR score					0.92	(0.59, 1.45)	0.74	(0.48, 1.13)	0.74	(0.48, 1.13)

Notes: estimates in all models are adjusted for age, sex, survey year, years of education, being in paid employment, and total net household income. LR = left-right self-placement.

Model 2 estimated the relationship between political regimes and self-rated health without controlling for individual political ideology. Respondents living in Christian conservative, liberal, or former Mediterranean dictatorship countries did not report poorer health than respondents from social democratic countries (respectively OR 1.05, 95% CI 0.70–1.63; OR 0.99, 95% CI 0.58–1.70; OR 1.00, 95% CI 0.61–1.65). In contrast, respondents from Eastern Europe, and especially people living in former Soviet republics, reported significantly poorer health than individuals in social democratic countries (OR 1.66, 95% CI 1.05–2.61 and OR 2.29, 95% CI 1.39–3.78, respectively).

In Model 3, we included the country aggregate political ideology measure, without controlling for individual ideology and political regimes. The results show that aggregate political ideology is not associated with reporting poor health (OR 0.92, 95% CI 0.59–1.45). Including individual political ideology and ecological political ideology measures simultaneously in Model 4 did not produce different results. The only exception was that Eastern Europeans no longer reported significantly poorer health than respondents from social democratic countries. Model 4(a) demonstrates that using the categorical measure of political ideology instead of the continuous version yielded similar patterns. As a sensitivity analysis, we re-estimated our models including individual political ideology using country-specific standardized z-scores for left-right self-placement. The results (shown in **Table S1**) demonstrate that using country-specific standardized z-scores for individual political ideology lead to the same conclusion in terms of patterns and effect size.

To examine the possibility that individual and ecological political ideology interact, we included cross-level interaction terms between individual political ideology and political regime and between individual political ideology and aggregated political ideology, separately, in supplemental models. The results (which are presented in **Table S2**) demonstrated that the association between left-right self-placement and self-rated health is strongest in social democratic countries (OR 0.92, 95% CI 0.89–0.95). In contrast, the relationship was even slightly positive among former Mediterranean dictatorships. Finally, the association between individual ideology and reporting poor health is strongest in societies where the aggregated individual ideology is more strongly right-wing oriented.

## Discussion

Our findings confirm both associations between individual political ideology self-rated health and between political regimes and self-rated health that were found in earlier work on Europe and the US. Moreover, the main health gap in these results, between Eastern Europe/former Soviet republics and all Western European political regimes, and the small differences between Western European regimes largely correspond to findings from an earlier multilevel study based on the same data.[2], [6] Interestingly, simultaneously including individual and ecological measures of political ideology did not lead to different conclusions regarding the independent importance of both individual ideology and political regime. Hence, associations between political regimes and health that were found in earlier work cannot be attributed to the association between individual ideology and health. Our results, therefore, suggest that political ideology is influential to health through several pathways at both the individual and contextual levels.

Although several mechanisms may explain how political regimes influence individual health,[2], [6] it is unlikely that individual political ideology has a direct causal influence on health. People's evaluation of the political left-right spectrum incorporates both materialistic and non-materialistic values, and is, therefore, a general marker of political ideology and values.[12] Probably, political ideology taps a broad range of values and beliefs (e.g., civic engagement, religiosity, and feelings of individual responsibility), which appear to benefit people's health. Additionally, the extent to which individual political ideology is associated with self-rated health varies between political regimes although the variation across some regimes was not statistically significant. Our finding that the association is strongest in social democratic countries deserves further exploration. Finally, the association could be due to reverse causality, meaning that good health would lead to conservative viewpoints. This would require truly longitudinal data on health and political ideology, which to our knowledge does not exist yet.

Although self-rated health is an often-used and validated indicator of overall mortality and morbidity, using ‘objective’ indicators of health would have been preferable. Unfortunately, the data did not include objective health markers. Therefore, possible reporting heterogeneity may partly account for the results. Additionally, although the political regime classification strongly resembles categorizations used in earlier work,[2], [6] the classification is subject to debate.[2], [6] As **Table S3** demonstrates, the signature of a country's political regime does not necessarily correspond to the political ideology of its residents (e.g., the percentage of individuals with rightwing ideologies is highest in the highly progressive social democratic regimes). Clearly, political regimes represent more than merely a simple sum of individual values and preferences. Using a different measure of ecological political ideology (i.e., a country level aggregation of individual ideology) to tap other mechanisms allowed us to address this issue. Although countries' aggregate political ideology did not appear to have a direct association with reporting poor health, our results suggest that the dominant ideology in people's living environment may determine to what extent individual political ideology translates into health problems.

It should be noted that regime typologies have been criticized for not shedding light on the exact mechanisms through which political regimes influence health, and for ignoring differences within regime types.[2], [6] However, the advantage of the present regime classification is that it is largely similar to the classifications most prominently used in earlier work. Therefore, deviating from this classification would be problematic for the comparability of our study with the existing literature. Finally, the present classification accounts for the dominant political ideology during the last few decades and not just the governmental ideology at the time of the survey. It is plausible that recent political history in many European countries (e.g., military dictatorships in Southern Europe and communism) has had a lasting impact on those countries' residents.

We should note that even though our analysis controlled for three commonly used dimensions of SES (*i.e.*, years of education, being in paid employment, and total net household income), the potential measurement error in these variables as well as unobserved dimensions of SES might still explain association between political ideology and health. We have acknowledged this possibility in the revised submission. At the same time, our reasons for a cautious confidence in our findings is that including the observed SES did not attenuate the individual level association between political ideology and health in any substantial manner. If there was substantial attenuation that would have increased the possibility of unobserved socioeconomic confounding even if the residual association was statistically significant.

Finally, the data documentation on the European Social Surveys (ESS) does not include information on selective non-response by respondents' political ideology, which could potentially account for the observed patterns. It however seems unlikely that this type of non-response could be driving our findings. For instance, the ESS is designed to be nationally representative and have relatively high overall response rates as compared to other survey data (about 60% on average). It should also be noted that if right winged individuals are more likely to take part in the survey this should have been evident in the distribution of this variable. However, we do not find evidence for this either (see **Table 2**, and **Table S3**).

In summary, this study shows that although both individual political ideology and contextual political regime are independently associated with individuals' self-rated health, accounting for SES and other demographic characteristics, individual political ideology appear to be more strongly associated with self-rated health than political regime. Further research is required to elucidate the mechanism through which political ideology at the individual and contextual level could influence health outcomes and behaviors among individuals.

## Supporting Information

Table S1 Results of age-, sex-, and SES-adjusted binary logistic, multilevel models, displaying odds-ratios (OR) and 95% confidence intervals (CI) for reporting poor health by individual political ideology (with country-specific standardized z-scores), political regime group, and aggregate political ideology in the 2002/04/06 European Social Survey. Note: all estimates are adjusted for age, sex, survey year, years of education, being in paid employment, and total net household income.
(0.04 MB DOC)

Table S2 Results of age-, sex-, and SES-adjusted binary logistic, multilevel models, displaying odds-ratios (OR) and 95% confidence intervals (CI) for cross-level interactions between individual political ideology (continuous and categorical) and political regime group and between individual political ideology (continuous and categorical) and aggregate political ideology, separately, on reporting poor health in the 2002/04/06 European Social Survey. Note: all estimates are adjusted for age, sex, survey year, years of education, being in paid employment, and total net household income.
(0.08 MB DOC)

Table S3 Left, middle, and right self-placement by country and by political regime in the European Social Survey (2002/04/06). Note: ‘Left’ = 0–3 on the left-right self-placement scale, ‘Middle’ = 4–6, and ‘Right’ = 7–10.
(0.06 MB DOC)
