# Supplementary material for: Political Regimes, Political Ideology, and Self-Rated Health in Europe: A Multilevel Analysis
Source: PLoS One. 2010 Jul 22;5(7):e11711. doi: 10.1371/journal.pone.0011711 (PMC2908625; doi:10.1371/journal.pone.0011711)
Supplement: Table S1 — Results of age-, sex-, and SES-adjusted binary logistic, multilevel models, displaying odds-ratios (OR) and 95% confidence intervals (CI) for reporting poor health by individual political ideology (with country-specific standardized z-scores), political regime group, and aggregate political ideology in the 2002/04/06 European Social Survey. Note: all estimates are adjusted for age, sex, survey year, years of education, being in paid employment, and total net household income. (0.04 MB DOC) [file pone.0011711.s001.doc]

Table S1. Results of age-, sex-, and SES-adjusted binary logistic, multilevel models, displaying odds-ratios (OR) and 95% confidence intervals (CI) for reporting poor health by individual political ideology (with country-specific standardized z-scores), political regime group, and aggregate political ideology in the 2002/04/06 European Social Survey.

|  | **Model 1** | | **Model 2** | | **Model 3** | | **Model 4** | |
| --- | --- | --- | --- | --- | --- | --- | --- | --- |
| **Variables** | **OR** | **95% CI** | **OR** | **95% CI** | **OR** | **95% CI** | **OR** | **95% CI** |
| Left-right self-placement (country-specific z-scores) (LR) | 0.91 | (0.88 , 0.93) |  |  |  |  | 0.90 | (0.88 , 0.93) |
| Social democratic (ref.) |  |  | 1.00 |  |  |  | 1.00 |  |
| Christian conservative |  |  | 1.07 | (0.70 , 1.63) |  |  | 0.90 | (0.57 , 1.43) |
| Liberal |  |  | 0.99 | (0.58 , 1.70) |  |  | 0.97 | (0.58 , 1.63) |
| Former Mediterranean dictatorships |  |  | 1.00 | (0.61 , 1.65) |  |  | 0.88 | (0.53 , 1.46) |
| Eastern Europe |  |  | 1.66 | (1.05 , 2.61) |  |  | 1.42 | (0.89 , 2.29) |
| Former Soviet republics |  |  | 2.29 | (1.39 , 3.78) |  |  | 2.23 | (1.38 , 3.61) |
| National average LR score |  |  |  |  | 0.92 | (0.59 , 1.45) | 0.71 | (0.46 , 1.08) |

Note: all estimates are adjusted for age, sex, survey year, years of education, being in paid employment, and total net household income.
