# Supplementary material for: Political Regimes, Political Ideology, and Self-Rated Health in Europe: A Multilevel Analysis
Source: PLoS One. 2010 Jul 22;5(7):e11711. doi: 10.1371/journal.pone.0011711 (PMC2908625; doi:10.1371/journal.pone.0011711)
Supplement: Table S2 — Results of age-, sex-, and SES-adjusted binary logistic, multilevel models, displaying odds-ratios (OR) and 95% confidence intervals (CI) for cross-level interactions between individual political ideology (continuous and categorical) and political regime group and between individual political ideology (continuous and categorical) and aggregate political ideology, separately, on reporting poor health in the 2002/04/06 European Social Survey. Note: all estimates are adjusted for age, sex, survey year, years of education, being in paid employment, and total net household income. (0.08 MB DOC) [file pone.0011711.s002.doc]

Table S2. Results of age-, sex-, and SES-adjusted binary logistic, multilevel models, displaying odds-ratios (OR) and 95% confidence intervals (CI) for cross-level interactions between individual political ideology (continuous and categorical) and political regime group and between individual political ideology (continuous and categorical) and aggregate political ideology, separately, on reporting poor health in the 2002/04/06 European Social Survey.

|  | **Model 1** | | **Model 2** | | **Model 3** | | **Model 4** | |
| --- | --- | --- | --- | --- | --- | --- | --- | --- |
| **Variables** | **OR** | **95% CI** | **OR** | **95% CI** | **OR** | **95% CI** | **OR** | **95% CI** |
| Left-right self-placement (LR) | 0.92 | (0.89 , 0.95) |  |  | 1.12 | (0.95 , 1.33) |  |  |
| Left (ref.) |  |  | 1.00 |  |  |  | 1.00 |  |
| Middle |  |  | 0.66 | (0.56 , 0.79) |  |  | 2.19 | (0.87 , 5.48) |
| Right |  |  | 0.62 | (0.51 , 0.75) |  |  | 3.63 | (1.20 , 10.98) |
| Social democratic (ref.) | 1.00 |  | 1.00 |  |  |  |  |  |
| Christian conservative | 0.94 | (0.59 , 1.49) | 0.90 | (0.58 , 1.41) |  |  |  |  |
| Liberal | 0.71 | (0.39 , 1.29) | 0.77 | (0.43 , 1.37) |  |  |  |  |
| Former Mediterranean dictatorships | 0.63 | (0.37 , 1.10) | 0.73 | (0.43 , 1.24) |  |  |  |  |
| Eastern Europe | 1.32 | (0.81 , 2.15) | 1.44 | (0.90 , 2.32) |  |  |  |  |
| Former Soviet republics | 2.15 | (1.23 , 3.76) | 1.87 | (1.09 , 3.21) |  |  |  |  |
| National average LR score |  |  |  |  | 1.13 | (0.70 , 1.81) | 1.14 | (0.72 , 1.82) |
| LR * Christian conservative | 1.02 | (0.98 , 1.06) |  |  |  |  |  |  |
| LR * Liberal | 1.07 | (1.01 , 1.13) |  |  |  |  |  |  |
| LR * Former Mediterranean dictatorships | 1.09 | (1.04 , 1.15) |  |  |  |  |  |  |
| LR * Eastern Europe | 1.04 | (1.01 , 1.08) |  |  |  |  |  |  |
| LR * Former Soviet republics | 1.01 | (0.96 , 1.07) |  |  |  |  |  |  |
| Middle * Christian conservative |  |  | 1.25 | (1.01 , 1.55) |  |  |  |  |
| Middle * Liberal |  |  | 1.41 | (1.04 , 1.93) |  |  |  |  |
| Middle * Former Mediterranean dictatorships |  |  | 1.41 | (1.08 , 1.84) |  |  |  |  |
| Middle * Eastern Europe |  |  | 1.14 | (0.92 , 1.42) |  |  |  |  |
| Middle * Former Soviet republics |  |  | 1.46 | (1.10 , 1.93) |  |  |  |  |
| Right * Christian conservative |  |  | 1.14 | (0.89 , 1.46) |  |  |  |  |
| Right * Liberal |  |  | 1.38 | (0.98 , 1.94) |  |  |  |  |
| Right * Former Mediterranean dictatorships |  |  | 1.68 | (1.25 , 2.26) |  |  |  |  |
| Right * Eastern Europe |  |  | 1.20 | (0.94 , 1.54) |  |  |  |  |
| Right * Former Soviet republics |  |  | 1.03 | (0.74 , 1.43) |  |  |  |  |
| LR * National LR score |  |  |  |  | 0.97 | (0.94 , 1.00) |  |  |
| Middle * National LR score |  |  |  |  |  |  | 0.82 | (0.68 , 0.98) |
| Right * National LR score |  |  |  |  |  |  | 0.73 | (0.59 , 0.91) |

Note: all estimates are adjusted for age, sex, survey year, years of education, being in paid employment, and total net household income.
