# Supplementary material for: Political Regimes, Political Ideology, and Self-Rated Health in Europe: A Multilevel Analysis
Source: PLoS One. 2010 Jul 22;5(7):e11711. doi: 10.1371/journal.pone.0011711 (PMC2908625; doi:10.1371/journal.pone.0011711)
Supplement: Table S3 — Left, middle, and right self-placement by country and by political regime in the European Social Survey (2002/04/06). Note: ‘Left’ = 0–3 on the left-right self-placement scale, ‘Middle’ = 4–6, and ‘Right’ = 7–10. (0.06 MB DOC) [file pone.0011711.s003.doc]

Table S3. Left, middle, and right self-placement by country and by political regime in the European Social Survey (2002/04/06).

| **Country** | **% Left** | **% Middle** | **% Right** |
| --- | --- | --- | --- |
| Social democratic | 19.3 | 48.3 | 32.4 |
| Denmark | 16.0 | 51.3 | 32.7 |
| Finland | 13.0 | 50.2 | 36.8 |
| Norway | 21.7 | 48.6 | 29.6 |
| Sweden | 25.3 | 43.9 | 30.8 |
| Christian conservative | 24.6 | 55.6 | 19.8 |
| Austria | 23.5 | 62.1 | 14.4 |
| Belgium | 23.0 | 57.1 | 19.9 |
| France | 28.8 | 48.6 | 22.6 |
| Germany | 28.9 | 58.7 | 12.4 |
| Iceland | 23.0 | 48.9 | 28.2 |
| Italy | 29.9 | 51.0 | 19.1 |
| Luxembourg | 19.6 | 58.5 | 21.5 |
| Netherlands | 20.7 | 51.6 | 27.8 |
| Switzerland | 22.7 | 55.8 | 21.5 |
| Liberal | 16.5 | 60.5 | 23.0 |
| Ireland | 12.4 | 65.3 | 22.2 |
| Israel | 30.6 | 31.1 | 38.3 |
| United Kingdom | 15.2 | 66.5 | 18.3 |
| Former Mediterranean dictatorships | 24.2 | 51.9 | 24.0 |
| Cyprus | 28.5 | 38.1 | 33.4 |
| Greece | 13.7 | 54.4 | 31.9 |
| Portugal | 24.3 | 53.2 | 22.4 |
| Spain | 33.6 | 51.6 | 14.8 |
| Eastern Europe | 23.8 | 51.0 | 25.2 |
| Bulgaria | 38.7 | 43.3 | 18.1 |
| Czech Republic | 22.1 | 46.0 | 31.9 |
| Hungary | 25.7 | 50.0 | 24.3 |
| Poland | 18.4 | 53.2 | 28.4 |
| Slovenia | 25.3 | 55.9 | 18.8 |
| Slovakia | 26.1 | 50.9 | 23.0 |
| Former Soviet republics | 16.8 | 56.0 | 27.2 |
| Estonia | 19.6 | 56.6 | 23.8 |
| Latvia | 12.5 | 54.4 | 33.1 |
| Russian Federation | 15.5 | 64.0 | 20.5 |
| Ukraine | 19.2 | 48.1 | 32.7 |
| Total (pooled) | 21.9 | 53.4 | 24.7 |

Note: ‘Left’ = 0-3 on the left-right self-placement scale, ‘Middle’ = 4-6, and ‘Right’ = 7-10.
